# Editorial: Pharmacokinetic evaluation and modeling of clinically significant drug metabolites, Volume II

**DOI:** 10.3389/fphar.2022.1087988

**Published:** 2023-01-04

**Authors:** Momir Mikov, Maja Đanić, Slavica Lazarević, Nebojša Pavlović, Bojan Stanimirov, Hani Al-Salami, Armin Mooranian

**Affiliations:** ^1^ Department of Pharmacology, Toxicology and Clinical Pharmacology, Faculty of Medicine, University of Novi Sad, Novi Sad, Serbia; ^2^ Department of Pharmacy, Faculty of Medicine, University of Novi Sad, Novi Sad, Serbia; ^3^ Department of Biochemistry, Faculty of Medicine, University of Novi Sad, Novi Sad, Serbia; ^4^ The Biotechnology and Drug Development Research Laboratory, Curtin Medical School & Curtin Health Innovation Research Institute, Curtin University, Bentley, WA, Australia; ^5^ Hearing Therapeutics Department, Ear Science Institute Australia, Queen Elizabeth II Medical Centre, Nedlands, WA, Australia

**Keywords:** drug metabolism, *in vitro*-*in vivo* extrapolation, PBPK absorption modeling, pharmacokinetics, bioequivalance

Comprehensive *in vivo* evaluation of a drug pharmacokinetics in preclinical stage can be expensive and time-consuming. Furthermore, using *in vivo* animal data may cause unreliable predictions of bioavailability in humans. Therefore, mathematical models that use inputs from *in vitro* assays have emerged as powerful tools to predict the pharmacokinetic behavior of drugs in the human body (Lin and Wong, 2017; Davanço et al., 2020). In the last decade, the application of the *in vitro-in vivo* correlation (IVIVC) for pharmaceutical dosage forms has become a center of attention of pharmaceutical industry, regulatory sectors and academia to achieve a more rational and assertive drug development flow (Jones et al., 2015). Physiologically based pharmacokinetic modeling (PBPK modeling) is a mathematical predictive IVIVC approach, which can quantitatively predict drug pharmacokinetics in human or animal by extrapolating *in vitro*, *in situ*, and *in silico* drug-dependent parameters. In comparison to the traditional deconvolution IVIVC approach, it is characterized by flexibility to incorporate physiological absorption processes (such as transporter involvement or regional-dependent intestinal drug absorption) as well as compound-specific parameters such as enzyme maximum rate of metabolism (Reddy et al., 2021). Nevertheless, application of such correlations encounters challenges in the cases of drugs which undergo substantial metabolism. Recent examples in the literature demonstrated relevance of drug metabolites and their consideration in the drug-drug interactions and bioequivalence evaluation (Gu et al., 2018; Mircioiu et al., 2019; Posada et al., 2020). Modelling of both parent and metabolite pharmacokinetics may be essential in some situations that cannot be solved without evaluation of metabolites.

In the frame of this Research Topic, contributions have been received concerning development of different PBPK models for sophisticated mechanistic applications such as the evaluation of bioequivalence, the prediction of plasma concentration profiles in special populations and the drug-drug interactions.

Also, this Research Topic benefited from important results regarding recommendations for personalized treatment in the case of drugs which undergo extensive metabolism and have highly variable responses. Below are examples, representative contributions to this literature body.

*Zhang et al.* have shown on the example of different dexketoprofen trometamol formulations how the PBPK model, as a convenient tool to evaluate the dissolution, permeability, and gastric emptying time which affect the pharmacokinetic parameters of the drug, can be applied to the risk assessment of biowaivers to reduce the possibility of waiver failure (Zhang et al.). In this study, however, it was concluded that the influence of excipients on liquid gastric emptying as a sensitive factor needs to be further studied when rapidly-eliminating drugs belonging to the Biopharmaceutics Classification System (BCS) class I are biowaived. These results open new avenues of research in this field.

Accordingly, the significance of building PBPK models for the new pharmaceutical formulations has been emphasized in the study by Zhang et al. Considering the extremely low aqueous solubility of antiviral drug ST-246, its ternary complex with meglumine and hydroxypropyl-β-cyclodextrin has been developed for intravenous administration. PBPK model was built and validated based on comprehensive preclinical studies and available knowledge to optimize the dose regimen of intravenous infusion for the treatment of severe smallpox, which may facilitate the clinical translation of this novel pharmaceutical formulation.

Additionally, a growing emphasis on the application of PBPK modeling has been placed to quantitatively evaluate the potential risks of drug-drug interactions. Also, due to the fact that most drugs are primarily eliminated *via* liver and kidneys, the effect of the impairment of these organs on drug exposure, that is, drug–disease interaction, has also been pointed out. Accordingly, Liu et al. have developed a PBPK model of apatinib to provide mechanistic insights for understanding the potential risk of external and internal factors on apatinib pharmacokinetics, providing the reasonable dosing recommendations (Liu et al.).

Dosing recommendations were also created for the lipopeptide antibiotic daptomycin in the study by Ye et al. The authors used PBPK modeling to evaluate the pharmacokinetic changes of daptomycin in the population of children with renal impairment and, after evaluating the drug’s pharmacodynamics and adverse effects, they obtained the recommended doses of daptomycin for pediatric patients with renal insufficiency. These results fill the gap of experimental data on this drug pharmacokinetics in renal impaired children which could lead to tailored regimens for the vulnerable population.

*Miljković et al.* investigated the influence of age, but also of gender and body mass index on the pharmacokinetics of itraconazole, an antifungal agent with highly variable pharmacokinetics, in healthy subjects (Miljković et al.). They showed that only gender had a significant effect on area under the curve (AUC) as a measure of total systemic exposure to the drug, but genetic polymorphisms of selected Cytochromes P450 (CYP) enzymes and gender differences in drug pharmacokinetics could not be related. These results may suggest that the highly variable pharmacokinetics of itraconazole can be largely attributed to other factors, such as the contribution of intestinal metabolic enzymes and transporters, as well as gut microbial enzymes.

The need for new mechanistic models to investigate the transport of solutes at the organ, tissue, cell or membrane level was emphasized in the mini-review by King et al. The authors concluded that the advanced mechanistic models of transporter-dependent flux with more reliable and efficient predictions can be developed when integrated with *in vitro* experiments, which requires a close collaboration between modelers, materials scientists, and biologists.

Furthermore, this Research Topic of articles contains a mini-review which provided novel insights into the current evidence on microbiota-mediated metabolism of azathioprine, a prodrug which is metabolized in its active form in tissue and erythrocytes (Lazarević et al.). They proposed concepts for the identification of gut bacteria species involved in metabolism of azathioprine that could aid in the clinical implementation of novel tools for personalized thiopurine therapy of inflammatory bowel disease.

Also, a non-compartmental analysis for a comparative bioavailability study was published within this Research Topic. Firstly, *Rizea-Savu et al.* have developed and validated a HPLC-MS/MS analytical method for simultaneous determination of dexamfetamine and its prodrug, lisdexamfetamine, in human plasma (Rizea-Savu et al.). They applied the new method for the first comparative bioavailability study of lisdexamfetamine oral solution *versus* capsules and suggested that biotransformation of lisdexamfetamine by erythrocytes is the main process controlling the rate of dexamfetamine delivery. This paper illustrates how important the consideration of active metabolites is for bioequivalence evaluation.

The articles included in this Research Topic provide new proposals for biowaiver risk assessment and dosing recommendations based on PBKP model and classical pharmacokinetic models. In addition, the mini-reviews serve as a firm basis for future research on innovative tools for pharmacokinetic predictions and tools for the improvement of precision medicine.
